# The trisaccharide raffinose modulates epidermal differentiation through activation of liver X receptor

**DOI:** 10.1038/srep43823

**Published:** 2017-03-07

**Authors:** Tae-Young Na, Gyeong-Hwan Kim, Hyeon-Jeong Oh, Min-Ho Lee, Yong-Hyun Han, Ki Taek Kim, Ji-Su Kim, Dae-Duk Kim, Mi-Ock Lee

**Affiliations:** 1 College of Pharmacy, Seoul National University, Seoul 08826, Korea; 2 Research Institute of Pharmaceutical Sciences, Seoul National University, Seoul 08826, Korea; 3 Bio-MAX institute, Seoul National University, Seoul 08826, Korea

## Abstract

The epidermal barrier function requires optimal keratinocyte differentiation and epidermal lipid synthesis. Liver X receptor (LXR) α and β, are important transcriptional regulators of the epidermal gene expression. Here, we show that raffinose, a ubiquitously present trisaccharide in plants, activated the transcriptional activity of LXRα/β, which led to the induction of genes required for keratinocyte differentiation such as involucrin and filaggrin, and genes involved in lipid metabolism and transport including SCD1 and ABCA1 in both HaCaT and normal human epidermal keratinocytes. Raffinose induced the expression of JunD and Fra1, and their DNA binding in the AP1 motif in the promoters of involucrin and loricrin. Interestingly, LXR bound the AP1 motif upon raffinose treatment, and conversely, JunD and Fra1 bound the LXR response element in promoters of LXR target genes, which indicates the presence of a postive cross-talk between LXR and AP1 in the regualtion of these genes. Finally, the effect of raffinose in epidermal barrier function was confirmed by applying raffinose in an ointment formulation to the skin of hairless mice. These findings suggest that raffinose could be examined as an ingredient in functional cosmetics and therapeutic agents for the treatment of cutaneous disorders associated with abnormal epidermal barrier function.

A major function of the epidermis is to provide both a mechanical and permeability barrier between the external environment and the internal milieu of the skin. The permeability barrier function provided by the stratum corneum comprises the corneocytes which form the cornified layers, and patterned lipid lamellae which localize to the extracellular spaces between corneocytes^1^,^2^. The transition of keratinocytes from the basal layer to the stratum corneum is accompanied by many changes in the expression of genes including those encoding keratins and cornified envelope-associated cross-linking proteins such as involucrin and loricrin^3^. Epidermal lipids such as cholesterol, phospholipids, and glucosylceramides are synthesized during the differentiation of keratinocytes and are stored in epidermal lamellar bodies, which appear in the upper stratum spinosum layer^4^. The epidermal barrier is constantly regenerated from differentiating keratinocytes, and abnormalities in this process are associated with a variety of skin diseases such as ichthyosis, psoriasis, and atopic dermatitis^1^,^3^,^5^. Both keratinocyte differentiation and epidermal lipid synthesis are regulated by several nuclear receptors including retinoic acid receptors, peroxisome proliferator-activated receptors (PPARs), and liver X receptors (LXRs)^6^,^7^.

LXRα and LXRβ are lipid-activated transcription factors expressed in the epidermis and are important regulators of epidermal barrier function of the skin. The activation of both isoforms leads to stimulation of keratinocyte differentiation, epidermal lipid synthesis, and anti-inflammatory responses in skin cells^7^,^8^. Treatment with LXR activators such as 22(*R*)-hydroxycholesterol, TO901317, or GW3965 accelerate the formation of the epidermal permeability barrier and stimulate epidermal differentiation in the murine epidermis^9^,^10^. The LXR-induced epidermal function is accompanied by increased expression of genes involved in both early differentiation (e.g., involucrin) and late differentiation (e.g., loricrin, filaggrin, and transglutaminase (1). This increased gene expression is regulated by the activating protein 1 (AP1) family proteins in normal human epidermal keratinocytes (NHEKs)^11^,^12^. A genome-wide cistrome study revealed the coordination between LXR and AP1 signaling through their nearby DNA binding sites^13^. In addition, LXR induces the expression of genes that are involved in lipid metabolism and transport in keratinocytes. Upon topical application, LXR agonists augment epidermal lipid synthesis in murine skin, presumably by inducing expression of the ATP-binding cassette (ABC) family of lipid transporters, lipogenic factors such as Srebp-1c and Fasn, and enzymes in the ceramide biosynthetic pathway including ceramide synthase and sphingomyelin phosphodiesterase^14^,^15^,^16^,^17^. Transcription of aquaporin-3 (AQP3), a gene encoding a transporter of water and glycerol, is stimulated by LXR activators in cultured human keratinocytes^18^. Thus, adequate stimulation of LXRs is considered to be essential for improving epidermal barrier function and thereby maintaining healthy skin homeostasis.

Bioactive oligosaccharides, usually containing 3–10 sugar moieties, can be isolated from natural sources and exhibit various biological activities, which are strongly affected by their chemical structures^19^. In the skin, oligosaccharides have beneficial effects such as influencing water-holding properties of the stratum corneum, anti-inflammatory effect, sebum regulating functions, and antibacterial action^20^,^21^. Oligosaccharides are used as stabilizers and bulking agents in the formulation of cosmetics^19^,^22^. The raffinose family of oligosaccharides (RFOs) includes α-galactosyl derivatives of sucrose that are abundant in the plant kingdom and are found in a large variety of seeds such as legumes, lentils, peas, and beans^23^. The most common RFOs are the trisaccharide raffinose, tetrasaccharide stachyose, and pentasaccharide verbascose. Although the RFOs have been suggested as potential ingredients of cosmetics with moisturizing function for dry skin, the cellular, biological, and pathophysiological effects on the epidermal barrier have not been examined in detail^24^. Here, we report that raffinose activated transcriptional activity of LXR enhances epidermal barrier function through induction of genes such as involucrin, filaggrin, and AQP3.

## Results

### Raffinose is an activator of LXR

**Figure 1:**
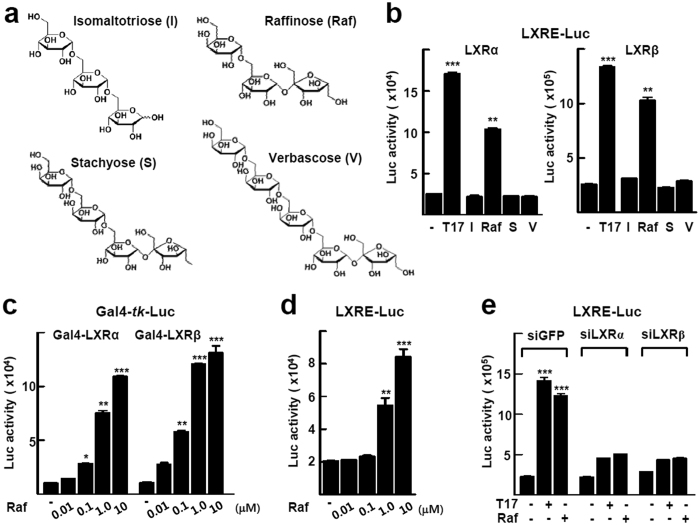
Raffinose activates transcriptional function of LXRα. (**a**) Chemical structure of isomaltotriose (I), raffinose (Raf), stachyose (S), and verbascose (V). **(b)** CV-1 cells were transfected with LXRE-Luc and expression vector for LXRα or LXRβ, and then treated with the indicated oligosaccharide or TO901317 (T17) at 1 μM for 24 h. **(c)** CV-1 cells were transfected with Gal4-*tk*-Luc and pGal4-LXRα or pGal4-LXRβ, and then treated with the indicated concentration of raffinose for 24 h. **(d)** HaCaT cells were transfected with LXRE-Luc and then treated with vehicle or the indicated concentration of raffinose for 24 h. **(e)** HaCaT cells were transfected with LXRE-Luc together with siGFP control, siLXRα, or siLXRβ. After 24 h of transfection, the cells were treated with 1 μM raffinose or 1 μM TO901317 for 24 h. The β-galactosidase activity was used to normalize luciferase activity. Data represent the means ± SEM of three independent experiments. *P < 0.05, **P < 0.01, and ***P < 0.001 compared with vehicle.

First, we used the luciferase reporter gene encoding LXRE to test whether RFOs activate the transcriptional activity of LXR (Fig. 1a). Treatment with 1 μM raffinose activated both LXRα- and LXRβ-mediated reporter activities, which were about 60 and 75% of that induced by 1 μM TO901317 treatment in CV-1 cells, respectively. However, other RFOs such as stachyose, verbascose, and a different form of trisaccharide isomaltotriose did not activate these activities (Fig. 1b). Raffinose induced the transactivation function of both Gal4DBD-LXRα and Gal4DBD-LXRβ on Gal4-*tk*-Luc in a dose-dependent manner in CV-1 cells, which indicated that the transactivation function of raffinose was LXR specific (Fig. 1c). Raffinose treatment activated LXRE-Luc without cotransfection of LXR in HaCaT cells, which suggested that endogenous LXR may function in response to raffinose in these cells (Fig. 1d). Consistently, suppression of the expression of both LXRα and LXRβ using small interfering RNA (siRNA) abolished the TO901317- or raffinose-induced reporter activities, which confirmed the LXR-dependent effect of raffinose (Fig. 1e). Raffinose did not induce the transcriptional activity of either PPARα or PPARγ (Supplementary Fig. S1).

### Raffinose regulates genes involved in keratinocyte differentiation and lipid metabolism

**Figure 2:**
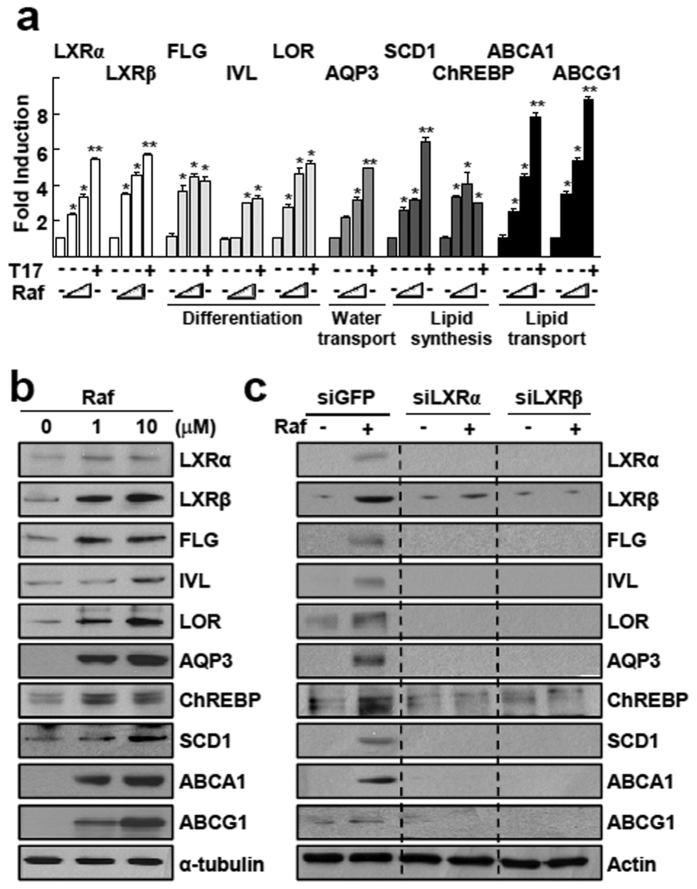
Raffinose stimulates transcription of genes involved in epidermal barrier function in HaCaT cells. (**a** and **b**) HaCaT cells were treated with vehicle, 1 μM or 10 μM raffinose (Raf), or 1 μM TO901317 (T17) for 24 h. Expressions of transcripts **(a)** and proteins **(b)** were analyzed by qRT-PCR or western blotting, respectively. FLG; filaggrin. IVL; involucrin, LOR; loricrin, AQP3; aquaporin3. **(c)** HaCaT cells were transfected with siGFP control, siLXRα, or siLXRβ, and then treated with 1 μM raffinose for 24 h. Expression of proteins was analyzed by western blotting. The original blots are shown in Supplementary Fig. S9.

Next, we examined whether raffinose could upregulate known LXR target genes that are involved in differentiation and lipogenesis in keratinocytes. Raffinose treatment increased both the protein and mRNA levels of LXRα and LXRβ (Fig. 2a,b). In a similar pattern, raffinose upregulated the expression of genes involved in keratinocyte differentiation such as filaggrin, involucrin, and loricrin; a gene involved in water transport, AQP3; LXR downstream lipogenic genes, stearoyl-CoA desaturase (SCD1) and carbohydrate-responsive element-binding protein (ChREBP); and genes involved in lipid transport such as the ABC family transporters, ABCA1 and ABCG1 (Fig. 2a,b). Knockdown of LXRα or LXRβ using siRNA abolished the induction of these LXR downstream target genes, which indicated that the effect of raffinose in keratinocytes was LXR dependent (Fig. 2c). Similarly, in NHEK cells, raffinose treatment increased the mRNA levels of LXRα and LXRβ, and their target genes (Supplementary Fig. S2).

**Figure 3:**
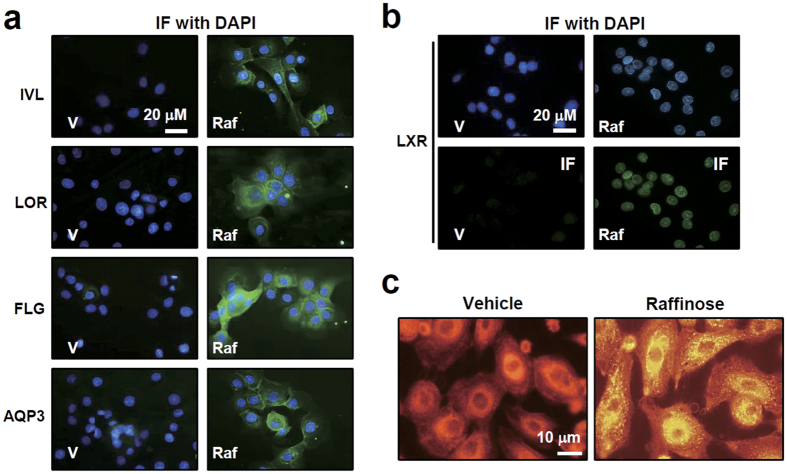
Raffinose induces expression of genes involved in keratinocyte differentiation and lipid accumulation in NHEKs. (**a** and **b**) NHEKs were treated with vehicle or 1 μM raffinose for 96 h. At the end of treatment, expression of the keratinocyte differentiation marker proteins and LXR were visualized by immunofluorescence (IF). 4,6-diaminidino-2-phenylindole (DAPI) was used to stain the nuclei. FLG; filaggrin. IVL; involucrin, LOR; loricrin, AQP3; aquaporin3. **(c)** NHEKs were treated with vehicle or 1 μM raffinose for 96 h and then lipid droplets were stained using Nile-red. 360 x magnification.

Raffinose induced morphological changes in NHEK cells produce flattened, enlarged, and squamous-like cells, which are typical features of differentiated keratinocytes (data not shown). Raffinose treatment increased the expression of keratinocyte differentiation markers such as involucrin, loricrin, filaggrin, and AQP3 in NHEKs (Fig. 3a). The expression of LXR was detected in the nucleus after raffinose treatment (Fig. 3b). Prolonged exposure to raffinose increased the number as well as size of lipid droplets in NHEKs and HaCaT cells when examined by Nile-red staining (Fig. 3c, Supplementary Fig. S3). Together, these results show that raffinose stimulated LXR-mediated keratinocyte differentiation and lipid accumulation.

### Raffinose induces a positive cross-talk between LXR and AP1 in the transcription of genes involved in keratinocyte differentiation

**Figure 4:**
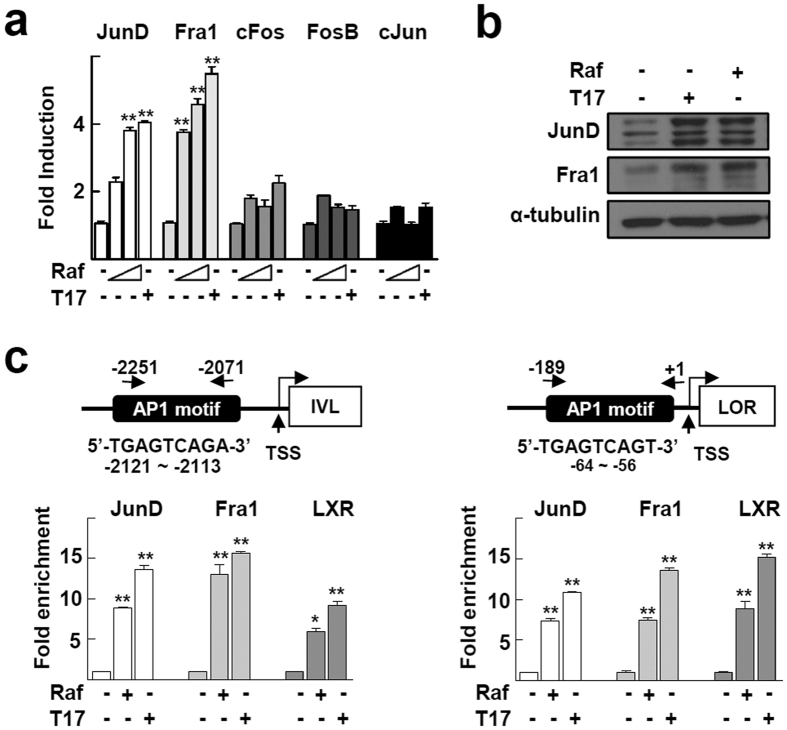
Raffinose induces expression as well as DNA binding of JunD and Fra1 in the promoters of invoucrin and loricrin. (**a**) HaCaT cells were treated with vehicle, 1 μM or 10 μM raffinose (Raf), or 1 μM TO901317 (T17) for 24 h. mRNA expression of the indicated AP1 components was analyzed by qRT-PCR. Numbers represent the means ± SEM of three independent experiments. **P < 0.01, and ***P < 0.001 compared with vehicle. **(b)** HaCaT cells were treated with vehicle, 1 μM raffinose, or 1 μM TO901317 for 24 h. Expression of JunD and Fra1 protein was analyzed by western blotting. The original blots are shown in Supplementary Fig. S9. **(c)** Schematic representation of AP1 response element in the promoters of involucrin (IVL) and loricrine (LOR) and primers used for ChIP assay (upper)^25^,^26^. HaCaT cells were treated with 1 μM raffinose or 1 μM TO901317 for 24 h. DNA fragments that were immunoprecipitated (IP) using the indicated antibodies were amplified by qPCR. *P < 0.05 and **P < 0.01 compared with vehicle treatment (lower).

We examined the mechanism by which raffinose can increase the transcriptional activity of LXR and the subsequent induction of downstream target genes. First, we examined whether raffinose can bind directly to either LXRα or LXRβ protein. A cell-free time-resolved fluorescence resonance energy transfer (FRET) assay showed that raffinose did not induce a signal from the ligand-dependent coactivator recruitment to either LXRα or LXRβ, which indicates that raffinose is not a ligand of LXRs (Supplementary Fig. S4). Because LXR agonists, such as oxysterols, increase the expression of the involucrin gene by inducing AP1 family proteins, we next examined the expression of the AP1 complex. Among the AP1 components tested, JunD and Fra1 were markedly induced at both the mRNA and protein level by raffinose treatment (Fig. 4a,b). Increase in the JunD protein level was detected at 14 h after raffinose treatment, which was similar to that of an LXR downstream target, ChREBP (Supplementary Fig. S5). A chromatin immunoprecipitation (ChIP) assay showed that raffinose increased DNA binding of JunD and Fra1 in the AP1 motifs present in the upstream promoters of involucrin and loricrin^25^,^26^. Interestingly, LXRs also bound to the AP1 sites after treatment with raffinose or TO901317 (Fig. 4c).

**Figure 5:**
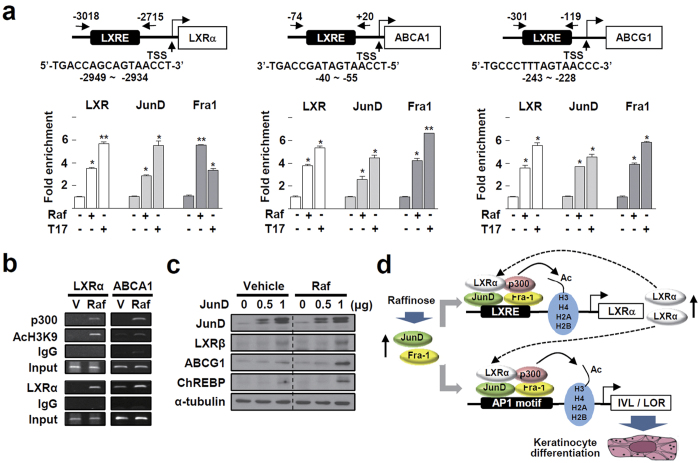
Raffinose induces DNA binding of LXR and AP1 in the LXREs in the promoters of LXR downstream genes. (**a**) Schematic representation of LXREs on the promoters of LXRα, ABCA1, and ABCG1 gene and primers used for ChIP assay (upper)^27^,^28^,^29^. HaCaT cells were treated with 1 μM raffinose (Raf) or 1 μM TO901317 (T17) for 24 h. DNA fragments that were immunoprecipitated using the indicated antibodies were amplified by qPCR (lower). *P < 0.05 and **P < 0.01 compared with vehicle treatment. Data represent the means ± SEM of three independent experiments. *P < 0.05 and **P < 0.01, compared with T17 treatment with empty vector. (**b**) HaCaT cells were treated with vehicle (V) or 1 μM raffinose (Raf) or for 24 h. DNA fragments that were immunoprecipitated using the indicated antibodies were amplified by PCR with specific primers for LXRα promoter or ABCA1 promoter as shown in panel **(a)**. **(c)** HaCaT cells were transfected with the indicated amount of expression vector encoding human JunD for 24 h, and treated with vehicle or 1 μM raffinose (Raf) or for 24 h. Expression of proteins was analyzed by western blotting. The original blots are shown in Supplementary Fig. S9. **(d)** Schematic representation of the proposed molecular mechanism of raffinose action in the keratinocyte differentiation.

It has been reported that about 80% of the LXR/retinoid X receptor binding sites in keratinocytes contain AP1 motifs in their vicinity. Therefore, we examined whether AP1 can affect the transcriptional function of LXR in the LXRE present in the promoter of LXR downstream genes. In accordance with the previous report, the well-characterized LXREs in the promoters of LXRα and ABCA1 overlapped by a consensus AP1 motiff 27,28,29. Interestingly, both raffinose and TO901317 induced DNA binding of LXR, JunD, and Fra1 in these LXREs (Fig. 5a). Raffinose also increased the binding of coactivator p300 to the LXREs and the acetylation status of histone 3 lysine 9 located near the elements, indicating that it activated transcription of the LXR target genes (Fig. 5b). When JunD was introduced exogenously to HaCaT cells, expression of LXR and its downstream genes such as ABCG1 and ChREBP was further enhanced by raffinose, demonstrating that AP1 enhances the transcriptional function of LXR (Fig. 5c). Similarly, JunD and Fra1 increased the TO901317-induced transcriptional function of both LXRα and LXRβ in a dose-dependent manner (Supplementary Fig. S6). Together, these results suggested that AP1 that induced by raffinose binds to LXRE or AP1 motif together with LXR, and then increased transcription of genes those are required for keratinocyte differentiation including LXRα, involucrine, and loricrine (Fig. 5d).

### Raffinose induces epidermal differentiation in the skin of hairless mice

**Figure 6:**
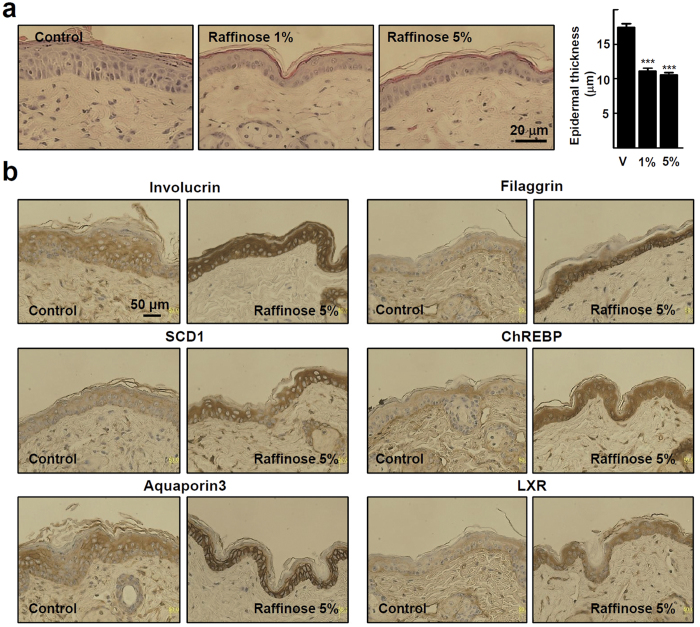
Raffinose increases expression of genes involved in keratinocyte differentiation in the skin of hairless mice. Hairless mice were treated topically twice a day for 4 days with 1 and 5% raffinose in ointment formulation. Control mice were treated with the base without raffinose. (**a**) Histological appearance of mouse skin was shown with H & E staining. 100 x magnification (left). Epidermal thickness of dorsal skin was measured at twelve sites in histological sections of three mice in each experimental group (right). Values are mean ± SEM (n = 3). *P < 0.05 compared with control. (**b**) Immunohistochemical staining for involucrin, filaggrin, SCD1, ChREBP, AQP3, and LXR in control and 5% raffinose-treated mouse skin. 100x magnification.

Finally, the effect of raffinose on the epidermal differentiation and proliferation was evaluated by applying raffinose in an ointment formulation to the skin of hairless mice. As shown in Fig. 6a, raffinose treatment reduced the epidermal thickness to about 60%. Raffinose treatment also caused a 50% decrease in the proliferating pool of epidermal keratinocytes, as determined by immunohistochemistry for proliferating cell nuclear antigen (Supplementary Fig. S7). Consistent with the results obtained from the *in vitro* studies, involucrin, filaggrin, SCD1, ChREBP, AQP3, and LXR stained strongly in the epidermis after application of raffinose (Fig. 6b). Taken together, these results demonstrate clearly that raffinose enhanced epidermal barrier function by inducing a positive cross-talk between LXR and AP1 in keratinocytes.

## Discussion

Oligosaccharides including RFOs are used as ingredients of cosmetic preparations because of their chemical properties as proper stabilizers, bulking agents, or moisturizers^21^,^22^. Raffinose, in particular, has been shown to promote the formation of lamellar structures in an artificial stratum corneum, which suggests that it offers advantages for recovery of skin barrier function^24^. However, the function of raffinose in keratinocyte differentiation and the associated molecular mechanisms are not known. Here, we found a newly identified mechanism of raffinose action in epidermis that activates an activation loop of LXR and AP1 to increase the expression of genes required for keratinocyte differentiation. Other trisaccharides such as isomaltotriose and other RFOs such as stachyose and verbascose did not activate LXR activity, which suggests that this type of raffinose action differs from the general effects of the group of oligosaccharides in the epidermis (Fig. 1).

Earlier studies have demonstrated that ligands of LXR, such as oxysterols and synthetic nonsteroidal agonists, increase both the expression and DNA binding of AP1 in the promoter of involucrin^11^,^12^. Interestingly, in our study, LXRs also bound to the AP1 motif and increased the expression of JunD and Fra1, which suggests the involvement of a positive cross-talk between LXR and AP1 in the activation of involucrin and loricrin (Fig. 4b,c). This cross-talk was also observed in the LXRE present in the promoters of LXR target genes such as ABCA1, ABCG1, and LXRα itself. A previous genome-wide cistrome analysis revealed an enrichment of AP1 motifs at a location adjacent to the LXR-RXR heterodimer binding sites^13^. Consistently, we found that the LXRE located in the promoter of LXRα or ABCA1 overlapped with an AP1 motif, 5′-TGACCAG-3′ or 5′-TGACCGA-3′, respectively. In addition to LXR, JunD and Fra1 also bound to the DNA region, which suggests that AP1 transactivates LXR in the LXR downstream target genes (Fig. 5). An inhibitor of the mitogen-activated protein kinase pathway, PD98059 decreased the raffinose- or TO901317-induced transcriptional activity of LXRα which supports the involvement of AP1 in LXR-mediated gene expression (Supplementary Fig. S8). Together, this newly identified transactivation mechanism of LXRα with AP1 on their cognate response elements seems to be essential for orchestrating the gene expression required for epidermal barrier function.

Raffinose, one of the most abundant water-soluble carbohydrates in plants, is a trisaccharide comprising galactose, glucose, and fructose^23^. In the human intestine, raffinose can be hydrolyzed to d-galactose and sucrose by α-galactosidase, an enzyme present in bacteria that is not found in the human digestive tract, and eventually fermented^19^. The mechanisms responsible for the cellular transport and intracellular metabolism of raffinose in keratinocytes are largely unknown. Previously, Seglen *et al*.^30^ used [^3^H]-labelled raffinose to show that raffinose accumulates in the cytoplasm of cultured rat hepatocytes^30^. Although a disaccharide, trehalose, is associated with one of the membrane glucose transporters, solute carrier 2 A, the involvement of raffinose in glucose transport has not been studied^31^. Further studies on the cellular uptake of raffinose and strategies to increase the absorption of raffinose in the skin will help to expand the spectrum of raffinose use as an ingredient of functional cosmetics. It was reported recently that raffinose is an activator of autophagy in human keratinocytes, which may help to ensure proper epidermal function^32^,^33^. Whether our finding of raffinose-induced LXR action plays a role in autophagy of keratinocytes and the relevance of this finding in other human diseases are interesting questions for future studies.

Abnormalities in the epidermal barrier function are associated with a variety of skin diseases such as ichthyosis, psoriasis, and atopic dermatitis^1^,^3^,^5^. The observation that LXRα gene knockdown in normal human keratinocytes simulated the genomic profile observed in psoriatic skin lesions suggests a role of LXR in the pathogenesis of this disease^34^. Importantly, activating ligands of LXR show beneficial effects against these diseases. For example, TO901317 exhibits potent antihyperplastic and anti-inflammatory activities in irritant contact dermatitis and acute allergic contact dermatitis murine models, and 22R-hydroxycholesterol, 25-hydroxycholesterol, and GW3965 display potent anti-inflammatory activity in both irritant and allergic contact models of dermatitis^35^,^36^. Therefore, raffinose may provide a new class of therapeutic agent for the treatment of cutaneous disorders in addition to its use in cosmetic products.

## Materials & Methods

### Cell culture and reagents

CV-1, a green monkey kidney cell line (ATCC CCL 70), and HaCaT, a spontaneously transformed human keratinocyte cell line^37^, were obtained from the American Type Culture Collection and the Cell Lines Service (CLS 300493, Eppelheim, Germany), respectively. Cells were maintained in Dulbecco’s modified Eagle’s medium (DMEM) containing 1.8 mM CaCl_2_ supplemented with 10% fetal bovine serum (FBS) at 37 °C in a humid atmosphere of 5% CO_2_. NHEKs were obtained from Lonza (Walkersville, MD) and maintained serum free in the keratinocyte basal medium KBM Gold (Lonza) containing 0.3 mM calcium supplemented with KGM-SingleQuot (Lonza). NHEKs were used for experiments within passage 2. Cells at 60 to 70% confluency were treated with vehicle, RFOs, or TO901317. Isomaltotriose and raffinose-series oligosaccharide family members including stachyose, verbascose, and d-(+)-raffinose·5H_2_O were purchased from Sigma-Aldrich (St Louis, MO). The synthetic LXR agonist T0901317 was purchased from Cayman Chemical (Ann Arbor, MI).

### Reporter gene assays

The luciferase reporters, LXRE-Luc and Gal4-*tk*-Luc, and the expression vectors for human LXRα were used as described previously. pCMV-mJunD was kindly donated by Dr Hongduk Yun (College of Medicine Seoul National University, Seoul, Korea)^38^,^39^. The Myc-tagged LXRβ or human Fra1 was constructed by inserting the corresponding cDNA into pCMV-Myc (Clontech, Palo Alto, CA). pGAL4-LXRα and pGAL4-LXRβ were constructed by inserting the full-length human LXRα or LXRβ cDNA into the expression vector containing the DNA-binding domain of yeast GAL4 as described previously^38^. All of the new constructs were verified by DNA sequencing. Transient transfection of the reporter gene and LXR expression vectors was performed as previously described^39^. Luciferase activity in whole-cell lysates was measured using a luminometer and normalized by β-galactosidase activity for transfection efficiency. For RNA interference, the siRNA duplexes for LXRα and LXRβ were transfected into cells as previously described (Table S1)^39^.

### Quantitative real-time polymerase chain reaction (qRT-PCR), western blotting, and ChIP assays

HaCaT cells were seeded in DMEM with 5% charcoal-stripped FBS, and the medium was changed to DMEM with 1% charcoal-stripped FBS for treatment of the testing materials to avoid any interference caused by steroid-like substances present in FBS. Isolation of total RNA, cDNA synthesis, and qRT-PCR were performed as previously described using specific primers as shown in Table S2. Western blotting was performed using specific antibodies against LXRα and LXRβ (PA1–332, Thermo Scientific, Waltham, MA), filaggrin (sc-66192, Santa Cruz Biotechnology, CA), SCD1 (sc-14719, Santa Cruz Biotechnology), loricrin (sc-51130, Santa Cruz Biotechnology), ABCA1 (ab18180, Abcam, Cambridge, UK), ABCG1 (ab52617, Abcam), involucrin (I9018, Sigma-Aldrich), ChREBP (NB400-135, Novus Biologicals, Littleton, CO), AQP3 (ab125219, Abcam), Actin (sc-1616, Santa Cruz Biotechnology), and α-tubulin (05-829, Millipore, Billerica, MA), as previously described^39^. ChIP assays were performed using antibodies against LXR (PA1-332, Thermo Scientific), JunD (sc-74, Santa Cruz Biotechnology), Fra1 (sc-28310, Santa Cruz Biotechnology), p300 (sc-585, Santa Cruz Biotechnology), and AcH3K9 (ab4441, Abcam). Immunoprecipitated DNA was amplified by PCR with specific primers as described previously (Table S2)^39^.

### Nile-red staining and immunocytochemistry

NHEK cells were seeded in 60-mm dishes and incubated overnight. After treatment with raffinose, the cells were washed twice with phosphate-buffered saline. The culture medium was changed every 48 h for raffinose treatment. Nile-red staining including fluorescence microscopy was performed as previously described^39^. Immunocytochemistry was performed essentially as previously described using specific antibodies against involucrin (I9018, Sigma-Aldrich), loricrin (sc-51130, Santa Cruz Biotechnology), filaggrin (sc-66192, Santa Cruz Biotechnology), AQP3 (ab125219, Abcam), and LXR (PP-PPZ0412-10, Perseus Proteomics, Tokyo, Japan)^39^.

### Animals and tissue preparation

Adult hairless mice (Skh:HR-1) 8 weeks of age (SLC Japan, Tokyo, Japan) were treated topically twice a day for 4 days with 1 and 5% raffinose dissolved in a mixture of PEG 400 and PEG 3350 at a weight ratio of 3:2 (0.1 ml applied to 2-cm^2^ area). Control hairless mice were treated with vehicle alone (n = 6). At the end of treatment, the dorsal skin was collected and the epidermis was isolated. Skin samples were fixed overnight in 4% formaldehyde and embedded in paraffin. For histological examination, a 5-μm section of Paraplast-embedded tissue was stained routinely with hematoxylin and eosin (H&E). Immunohistochemistry was examined with antibodies against involucrin (924401, BioLegend, San Diego, CA), filaggrin (sc-66192, Santa Cruz biotechnology), SCD1 (sc-14719, Santa Cruz biotechnology), ChREBP (NB400-135, Novus Biologicals, Littleton, CO), AQP3 (ab125219, Abcam), LXR (PP-PPZ0412-10, Perseus Proteomics), and PCNA (ab18197, Abcam). The experimental protocols were approved by the Seoul National university Institutional Animal Care and Use Committee (permission number SNU-150818-6) and all experiments were conducted according to the committee’s guidelines.

### Statistical analysis

Statistical analysis was performed using GraphPad Prism software (GraphPad Software, Inc., La Jolla, CA). Experimental values are expressed as the mean ± standard error of mean (SEM) of three independent experiments, unless otherwise indicated. Significance differences between two groups were identified using the nonparametric Mann–Whitney *U* test unless otherwise indicated. Mean differences were considered significant at *P* < 0.05.

## Additional Information

**How to cite this article:** Na, T.-Y. *et al*. The trisaccharide raffinose modulates epidermal differentiation through activation of liver X receptor. *Sci. Rep.* **7**, 43823; doi: 10.1038/srep43823 (2017).

**Publisher's note:** Springer Nature remains neutral with regard to jurisdictional claims in published maps and institutional affiliations.

## Supplementary Material

Supplementary Information
