# Supplementary material for: The trisaccharide raffinose modulates epidermal differentiation through activation of liver X receptor
Source: Sci Rep. 2017 Mar 7;7:43823. doi: 10.1038/srep43823 (PMC5339792; doi:10.1038/srep43823)
Supplement: Supplementary Information [file srep43823-s1.doc]

**SREP-16-31852A**

**SUPPLEMENTARY INFORMATIONS**

**The trisaccharide raffinose modulates epidermal differentiation**

**through activation of liver X receptor**

Tae-Young Na^1,*^, Gyeong-Hwan Kim^1,*^, Hyeon-Jeong Oh^1^,

Min-Ho Lee^1^, Yong-Hyun Han^1^, Ki-Taek Kim^1^, Ji-Su Kim^1^, Dae-Duk Kim^1,2^, & Mi-Ock Lee^1,2,3^

^1^College of Pharmacy, Seoul National University, Seoul 08826, Korea

^2^Research Institute of Pharmaceutical Sciences, Seoul National University, Seoul 08826, Korea

^3^Bio-MAX institute, Seoul National University, Seoul 08826, Korea

^*^These authors contributed equally to this work.

Corresponce and requests for materials should be addressed to: M.O.L. (E-mail: [molee@snu.ac.kr](mailto:molee@snu.ac.kr))

**I. SUPPLEMENTARY TABLES**

Table S1. siRNA sequences used for RNA interference

| **siRNA** | **Nucleotide sequence** | |
| --- | --- | --- |
| siLXRα | Sense  Antisense | 5'- GAGACAUCUCGGAGGUACATT-3'  5'- UGUACCUCCGAGAUGUCUCTT-3' |
| siLXRβ | Sense  Antisense | 5'- CGAGCUUUGCCGUGUCUGUTT-3'  5'- ACAGACACGGCAAAGCUCGTT-3' |

Table S2. Primer sequences used for qRT-PCR and ChIP

| **Gene** | **Nucleotide sequence** | |  |
| --- | --- | --- | --- |
| LXRα | Sense  Antisense | 5'- AGTGTCGGCTTCGCAAAT -3'  5'- AGAAGCATCACCTCGATCG-3' | qRT-PCR |
| LXRβ | Sense  Antisense | 5'- GAGTCACAGTCACAGTCGCAG -3'  5'- TCTCTAGCAGCATGATCTCGATA -3' | qRT-PCR |
| ChREBP | Sense  Antisense | 5'- ACAGCAACAAGACCGAGAAC -3'  5'- TGAAGGACTCAAACAGAGGC-3' | qRT-PCR |
| SCD1 | Sense  Antisense | 5'- ACTGGTGATGTTCCAGAGGA -3'  5'- GTTTCCATCTCCGGTTCTTT -3' | qRT-PCR |
| ABCA1 | Sense  Antisense | 5'- GGTGATGTTTCTGACCAATGTGA-3'  5'- TGTCCTCATACCAGTTGAGAGAC-3' | qRT-PCR |
| ABCG1 | Sense  Antisense | 5'- ACACCATCCCCACGTACCTA-3'  5'- GATGACCCCTTCGAACCCA-3' | qRT-PCR |
| filaggrin | Sense  Antisense | 5'- GGGAAGTTATCTTTTCCTGTC-3'  5'- GATGTGCTAGCCCTGATGTTG-3' | qRT-PCR |
| involucrin | Sense  Antisense | 5'- ACCCATCAGGAGCAAATGAAA-3'  5'- GCTCGACAGGCACCTTCTGGCA-3' | qRT-PCR |
| loricrin | Sense  Antisense | 5'-AGACCCAGCAGAAGCAGGCG -3'  5'-AGCAGAACTAGATGCAGCCG -3' | qRT-PCR |
| AQP3 | Sense  Antisense | 5'- GACAGAAGGAGCTGGTGTCC-3'  5'- ATGAGGATGCCCAGAGTGAC-3' | qRT-PCR |
| Fra1 | Sense  Antisense | 5'- GGAAGGAACTGACCGACTTCCT-3'  5'- CTTCCGGGATTTTGCAGATG-3' | qRT-PCR |
| JunD | Sense  Antisense | 5'- ATCGACATGGACACGCAGGAGC-3'  5'- CTCCGTGTTCTGACTCTTGAGG-3' | qRT-PCR |
| cFos | Sense  Antisense | 5'- GGATAGCCTCTCTTACTACCACTCACC-3'  5'- AATGAAGTTGGCACTGGAGACG-3' | qRT-PCR |
| JunB | Sense  Antisense | 5'- GGAACAGCCCTTCTACCACG-3'  5'- GGCTCGGTTTCAGGAGTTTG-3' | qRT-PCR |
| cJun | Sense  Antisense | 5'- CCTTGAAAGCTCAGAACTCGGAG-3'  5'- TGCTGCGTTAGCATGAGTTGGC-3' | qRT-PCR |
| β-actin | Sense  Antisense | 5'- CGTGGGCCGCCCTAGGCACCA-3'  5'- TTGGCTTAGGGTTCAGGGGGG-3' | qRT-PCR |
| LXRα  Promoter | Sense  Antisense | 5'- GGAACTGGAGTTCATAGCA-3'  5'- GCCTTTTCCAAGACCACA-3' | ChIP |
| involucrin  promoter | Sense  Antisense | 5'- CTCCCTTGCATACACACACA-3'  5'- GGTTAGCAGGGGCTCAGTAT-3' | ChIP |
| loricrin  promoter | Sense  Antisense | 5'- GCCCATCTCAAGAATGCCAA-3'  5'- GAAGATGCTGGCAATGTGAG-3' | ChIP |
| ABCA1  promoter | Sense  Antisense | 5'- TGCTTTCTGCTGAGTGACTGAACTAC-3'  5'- CAATTACGGGGTTTTTGCCG-3' | ChIP |
| ABCG1  promoter | Sense  Antisense | 5'- TTCTGTGGACAGGTACTAGGTG-3'  5'- TGTCTGTGTAATGCTACAGGGA-3' | ChIP |

**II. SUPPLEMENTARY FIGURES**


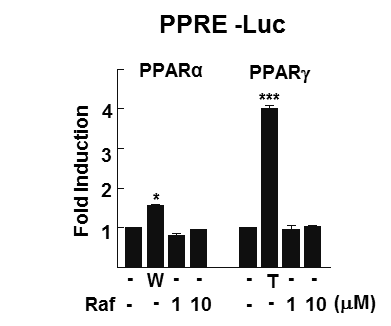


**Figure S1. PPARα and PPARγ are not induced by raffinose.** CV-1 cells were transfected with 50 μg LXRE-Luc and 1 ng PPARα or PPARγ. Transfected cells were treated with raffinose or 1μM Wy14643 (W) for PPARα and 1μM troglitazone (T) for PPARγ or 24 h. **P* < 0.05, ****P* < 0.001 compared with vehicle control (n=3). Statistical significance was performed using the Mann-Whitney *U* test.


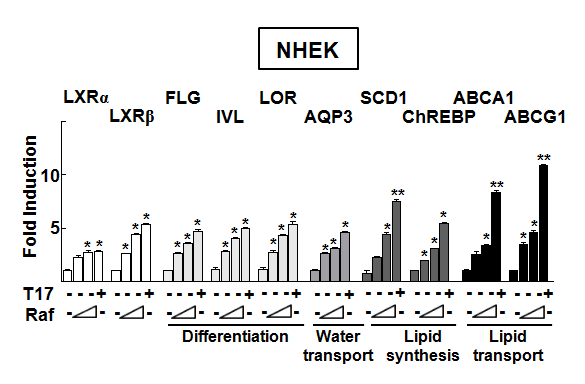


**Figure S2. Raffinose stimulates transcription of genes involved in epidermal barrier function in NHEKs.** NHEKs were treated with vehicle, 1 μM or 10 μM raffinose, or 1 μM TO901317 for 24 h. Total RNA was prepared and analyzed for expression of transcripts by qRT-PCR. **P* < 0.05, ****P* < 0.001 compared with vehicle control (n=3). Statistical significance was performed using the Mann-Whitney *U* test.

**
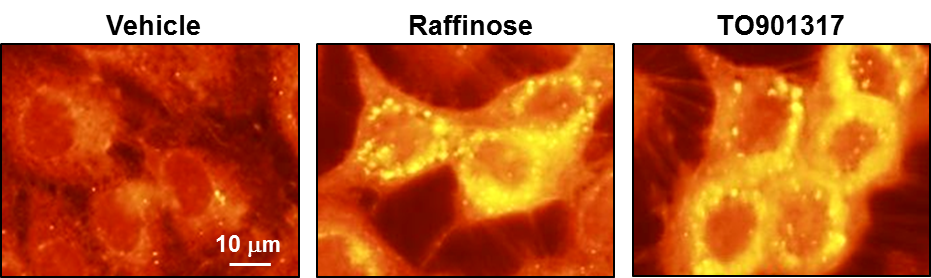
**

**Figure S3. Effect of raffinose on lipid accumulation in HaCaT cells.** HaCaT cells were treated with 1 μM raffinose for 6 days. At the end of treatment, lipid droplets were stained using Nile-red, and photographs were taken. 360x magnification.


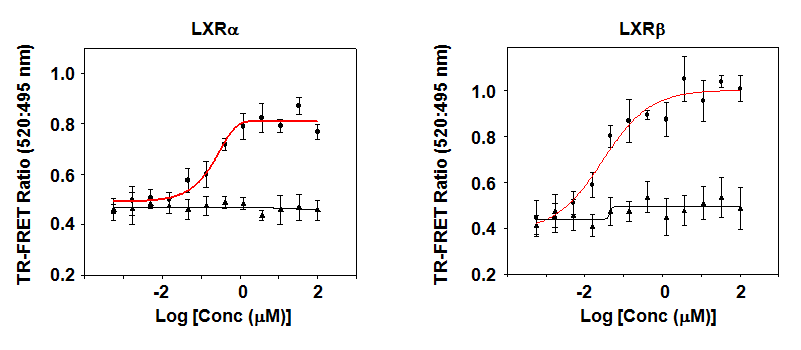


**Figure S4. Raffinose does not directly bind either LXRα or LXRβ.** TR-FRET assay was performed using Lanthascreen LXRα or LXRβ co-activator assay kit (Invitrogen, Madison, WI). Y-axis represents ratio of fluorescence intensity at 520 nm (signal) and at 495 nm (background). X-axis represents log scale of raffinose (black line) or TO901317 (red line) concentration. TO901317 was used as positive control. Data represents mean ± SD (n=4). The EC50 value of TO901317 was calculated as 30 nM and 29 nM for LXRα and LXRβ, respectively.


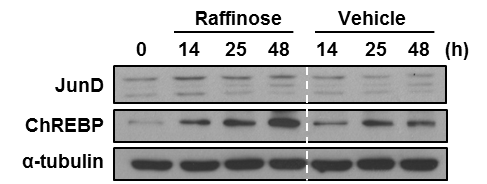


**Figure S5. Raffinose induces expression of JunD and ChREBP protein in HaCaT cells**. HaCaT cells were treated with 1 μM raffinose or vehicle for the indicated period. Expression of JunD and ChREBP, a downstream target of LXR, was analyzed by western blotting.


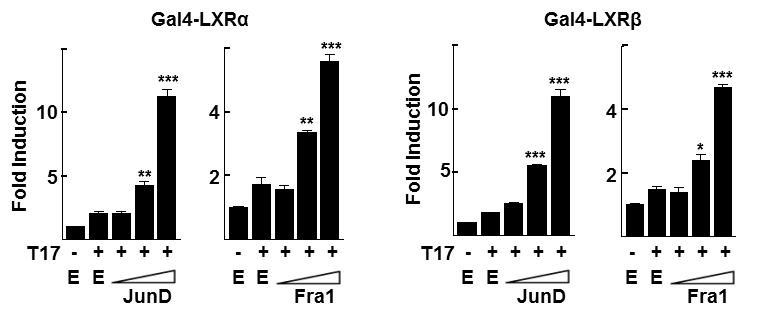


**Figure S6. JunD and Fra1 enhance the transcriptional activities of LXRs by TO901317.** CV-1 cells were transfected with Gal4-*tk*-Luc, pGal4-LXRα or pGal4-LXRβ, together with empty vector (E), or 10, 50, or 100 ng expression vector for JunD or Fra1. After 24 h of transfection, the cells were treated with 0.1 μM TO901317 (T17) for 24 h. The β-galactosidase activity was used to normalize luciferase activity. Data represent the means ± SEM of three independent experiments. ^*^*P*< 0.05 and ^**^*P*< 0.01, compared with T17 treatment with empty vector.


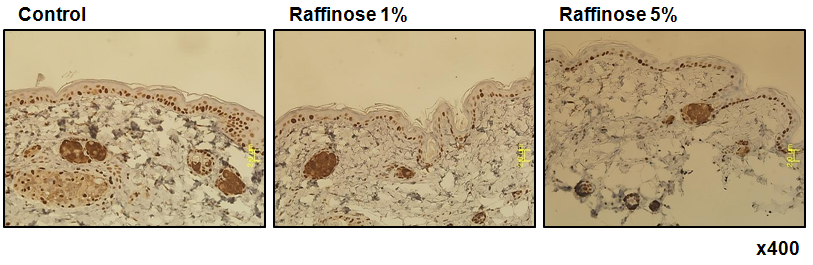


**
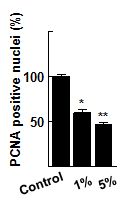
**

**Figure S7. Raffinose treatment decreases the number of PCNA positive nuclei in the skin of hairless mice.** Hairless mice were treated topically twice a day for 4 days with 1% and 5% raffinose in ointment formulation. Control mice were treated with the base without raffinose. Immunohistochemical staining for PCNA in control and raffinose-treated skin. 400x magnification (upper). The number of PCNA (brown) in epidermis was counted. Values are mean ± SEM based on counts in histological sections of three mice per each treatment group. **P* < 0.05 compared with control group (lower).


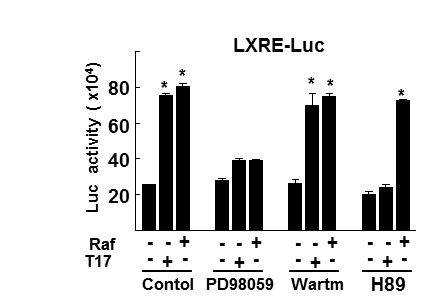


**Figure S8. PD98059 inhibits the raffinose-induced transactivation function of LXRα.** HaCaT cells were transfected with 50 μg LXRE-Luc and 1 ng LXRα. Transfected cells were treated with 1μM raffinose (Raf) or 1 μM TO901317 (T17) in the presence of 100 μM PD98059, 30 nM Wartmannin (Wartm), or 1 μm H89 for 24 h. **P* < 0.05 compared with control (n=3). Statistical significance was performed using the Mann-Whitney *U* test.


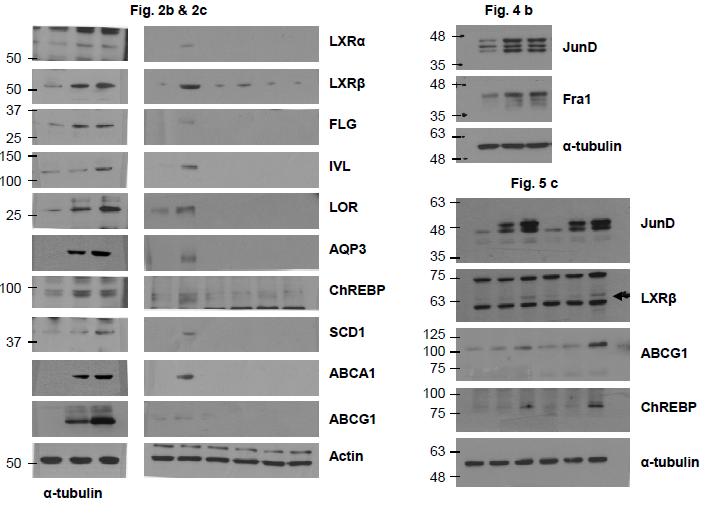


**Figure S9. The original images of blots presentd in the main figures.**
